# Identifying genes related to choriogenesis in insect panoistic ovaries by Suppression Subtractive Hybridization

**DOI:** 10.1186/1471-2164-10-206

**Published:** 2009-04-30

**Authors:** Paula Irles, Xavier Bellés, M Dolors Piulachs

**Affiliations:** 1 Institut de Biologia Evolutiva (UPF-CSIC), Passeig Marítim de la Barceloneta, 37. 08003 Barcelona, Spain; 2 Laboratorio Internacional de Cambio Global (LINCGlobal), PUC-CSIC, Departamento de Ecología, Facultad de Ciencias Biológicas, PUC, Alameda 340, PC 6513677, Santiago, Chile

## Abstract

**Background:**

Insect ovarioles are classified into two categories: panoistic and meroistic, the later having apparently evolved from an ancestral panoistic type. Molecular data on oogenesis is practically restricted to meroistic ovaries. If we aim at studying the evolutionary transition from panoistic to meroistic, data on panoistic ovaries should be gathered. To this end, we planned the construction of a Suppression Subtractive Hybridization (SSH) library to identify genes involved in panoistic choriogenesis, using the cockroach *Blattella germanica *as model.

**Results:**

We constructed a post-vitellogenic ovary library by SSH to isolate genes involved in choriogenesis in *B. germanica*. The tester library was prepared with an ovary pool from 6- to 7-day-old females, whereas the driver library was prepared with an ovary pool from 3- to 4-day-old females. From the SSH library, we obtained 258 high quality sequences which clustered into 34 unique sequences grouped in 19 contigs and 15 singlets. The sequences were compared against non-redundant NCBI databases using BLAST. We found that 44% of the unique sequences had homologous sequences in known genes of other organisms, whereas 56% had no significant similarity to any of the databases entries. A Gene Ontology analysis was carried out, classifying the 34 sequences into different functional categories. Seven of these gene sequences, representative of different categories and processes, were chosen to perform expression studies during the first gonadotrophic cycle by real-time PCR. Results showed that they were mainly expressed during post-vitellogenesis, which validates the SSH technique. In two of them corresponding to novel genes, we demonstrated that they are specifically expressed in the cytoplasm of follicular cells in basal oocytes at the time of choriogenesis.

**Conclusion:**

The SSH approach has proven to be useful in identifying ovarian genes expressed after vitellogenesis in *B. germanica*. For most of the genes, functions related to choriogenesis are postulated. The relatively high percentage of novel genes obtained and the practical absence of chorion genes typical of meroistic ovaries suggest that mechanisms regulating chorion formation in panoistic ovaries are significantly different from those of meroistic ones.

## Background

In medieval times, small animals were thought to be devoid of internal organs, their life being animated by a sort of magic or divine spirit. The first naturalist that clearly saw and reported the internal anatomy of an insect was the Bolognese Marcello Malpighi, in 1669. Among other organs, we ought to him the first astonishingly detailed description of the ovaries of the silkworm. At that time, the recently invented microscope was a key development for this change of observational scale, and soon others followed Malpighi's path. This led to recognize the high morphological diversity of insect ovaries.

To put a bit of order in that diversity, in 1874 A. Brandt [1,2] proposed a classification of insect ovaries into two categories, panoistic and meroistic. Panoistic defining ovaries in which all oogonia are eventually transformed into oocytes, and meroistic defining ovaries whose oogonia can derive into both oocytes and nurse cells. A further refinement was proposed by J. Gross in 1903 [1,2], who divided meroistic ovaries into polytrophic (nurse cells and oocytes alternating along the ovariole) and telotrophic (nurse cells localized in the germarium and connecting to oocytes by nutritive cords).

The panoistic type predominates among less modified insects, whereas meroistic are most common in more modified species, which suggested that ovaries evolved from panoistic to meroistic [1,2]. Studies facing the evolutionary transition from panoistic to meroistic have been largely based on morphological evidence. However, significant cues to reconstruct such a transition should be found at a molecular scale of observation. The problem is that insect molecular data is practically restricted to meroistic ovaries, and within this category, to considerably highly modified species, like the dipteran *Drosophila melanogaster *and the lepidopteran *Bombyx mori *[3,4]. If we aim at reconstructing the evolution from panoistic to meroistic ovaries, we should therefore gather data on the panoistic type at molecular level. The purpose of this work is contributing to this aim, using the cockroach *Blattella germanica*.

*B. germanica *is a hemimetabolous insect with reproduction mainly regulated by juvenile hormone (JH) [5]. In this cockroach, only one batch of basal oocytes mature synchronously in each gonadotrophic cycle, and after oviposition the eggs are deposited into an egg case or ootheca, which is transported by the female during the entire embryo development. In our laboratory, the first gonadotrophic cycle of *B. germanica *lasts eight days, and during this period the basal oocyte grows exponentially, showing a pattern parallel to that of circulating JH [6,7]. During the first gonadotrophic cycle three oogenesis stages can be distinguished: pre-vitellogenesis (from day 0 to day 3), vitellogenesis (from day 3 to 6) and choriogenesis (during day 7). While pre-vitellogenic, the basal oocyte is preparing for growth, JH is synthesised at very low rates, vitellogenin (Vg) synthesis in the fat body is just starting, and the intercellular spaces in the follicle are narrow. During vitellogenesis, JH and Vg show the highest rates of synthesis, basal oocytes grow exponentially, and the intercellular spaces in the follicle are large. Choriogenesis is characterized by rapidly decreasing rates of JH and Vg synthesis and by the formation of chorion layers, whereas the intercellular spaces in the follicle are narrow again [7].

Our general approach was to characterize at genomic level the main stages of oogenesis in the adult *B. germanica*, pre-vitellogenesis, vitellogenesis and choriogenesis. To begin with, we chose choriogenesis given that it is the most characteristic one and the best known stage in meroistic species [3,8]. To identify genes related to choriogenesis, a number of approaches could be potentially useful, namely differential display, cDNA macro and microarray and subtractive hybridization. Of these, Suppression Subtractive Hybridization (SSH) [9] has been successfully used for studying genes specifically involved in particular processes of insect development, like morphogenesis [10] and metamorphosis [11], or to obtain genes specifically expressed under certain physiological conditions [12]. These favourable results led us to choose the SSH approach for our purposes.

## Results

### General library statistics

In order to find genes expressed in the ovaries of adult *B. germanica *after the vitellogenic period (in particular during choriogenesis), we constructed a SSH library. The tester library was prepared with post-vitellogenic ovaries from 6- to 7-day-old females with chorionated oocytes, whereas the driver library was prepared with vitellogenic ovaries from 3- to 4-day-old adult females. cDNA sequencing from the SSH library resulted in 384 sequences, 126 of which were filtered as low quality reads or as ribosomal RNA, polyA^+ ^tails or vector sequences.

To identify unique sequences from the resulting 258 useful ESTs, we carried out an assemble using the CAP3 program against a database of 1,536 gene sequences from a cDNA library obtained from *B. germanica *adult ovary that had been generated in our laboratory. The ovarian cDNA library permitted the identification of the rather short fragments resulting from RsaI digestion during the SSH preparation. After assembling, the 258 ESTs were clustered into 34 unique sequences distributed into 19 contigs and 15 singlets, where contigs consisted in at least one EST coming from the SSH library together with a sequence of the cDNA library (Table 1). These 34 sequences were deposited in the EMBL gene bank database with accession numbers from [EMBL: FM253346] to [EMBL: FM253378], and [EMBL: FM210754] for yellow-g sequence.

**Table 1:** Checklist of the 34 unique sequences obtained by SSH in post-vitellogenic ovaries of *Blattella germanica*

**Sample**	ESTs *	**Length (bp)**	ORF **	**Best BLASTx**	**Species**	**E-value**	**GO (Molecular Function)**
BG30001	19	2702	Yes	No homology^a^	-	-	-
BG30002	2	794	Yes	No homology	-	-	-
**BG30003**	**52**	**1131**	**Yes**	**Cuticula protein-like**^a^	***Acyrthosiphon pisum***	**3.00E-07**	**Unknown**
**BG30004**	**2**	**347**	**Yes**	**Ribosomal protein L18A**	***Graphocephala atropunctata***	**9.00E-47**	**Structural constituent of ribosome**
BG30005	2	367	No	No homology	-	-	-
**BG30006**	**2**	**263**	**Yes**	**Ribosomal protein L24e**	***Nasonia vitripennis***	**1.00E-31**	**Structural constituent of ribosome**
BG30007	2	171	Yes	No homology	-	-	-
BG30008	3	279	Yes	No homology	-	-	-
BG30009	91	1172	Yes	No homology	-	-	-
BG30010	2	193	Yes	No homology	-	-	-
BG30011	2	179	Yes	No homology	-	-	-
**BG30012**	**5**	**195**	**No**	**EST LY_YIT_BG1527**	***Blattella germanica***	**2.00E -21**	**Unknown**
BG30013	2	231	No	No homology	-	-	-
BG30014	19	117	No	No homology	-	-	-
BG30015	2	768	Yes	No homology	-	-	-
BG30017	28	1561	Yes	No homology^a^	-	-	-
**BG30018**	**1**	**849**	**Yes**	**CG10407-PA-like**	***Tribolium castaneum***	**1.00E-23**	**Binding**
**BG30019**	**1**	**1616**	**Yes**	**Yellow-g**^a^	***Nasonia vitripennis***	**3.00E-29**	**Unknown**
**BG30020**	**Singlet**	**770**	**Yes**	**Baiser**	***Bombyx mori***	**4.00E-59**	**Unknown**
**BG30021**	**Singlet**	**523**	**Yes**	**Follicle cell protein 3c**^a^	***Nasonia vitripennis***	**7.00E-49**	**Unknown**
**BG30022**	**Singlet**	**317**	**Yes**	**Ribosomal protein L44e**	***Nasonia vitripennis***	**2.00E-40**	**Structural constituent of ribosome**
**BG30023**	**Singlet**	**191**	**Yes**	**Cabeza CG3606-PA-like**	***Tribolium castaneum***	**2.00E-06**	**Transcriptional activator activity**
**BG30024**	**Singlet**	**423**	**Yes**	**Ribosomal protein L36e**	***Nasonia vitripennis***	**2.00E-48**	**Structural constituent of ribosome**
**BG30025**	**Singlet**	**496**	**Yes**	**Ferritin 2**	***Acyrthosiphon pisum***	**5.00E-24**	**Ion binding**
**BG30026**	**Singlet**	**447**	**Yes**	**Troponin I**	***Aedes aegypti***	**3.00E-35**	**Protein binding**
**BG30027**	**Singlet**	**138**	**Yes**	**Cyclin B**	***Nasonia vitripennis***	**2.00E-06**	**Kinase regulator activity**
**BG30028**	**Singlet**	**136**	**Yes**	**Cathepsin-L**^a^	***Penaeus monodon***	**2.00E-08**	**Hydrolase activity**
BG30029	Singlet	168	No	No homology	-	-	-
BG30031	Singlet	318	Yes	No homology	-	-	-
BG30032	Singlet	150	Yes	No homology	-	-	-
BG30033	Singlet	146	Yes	No homology	-	-	-
BG30036	Singlet	394	Yes	No homology	-	-	-
BG30037	Singlet	348	Yes	No homology	-	-	-
BG30038	Singlet	306	No	No homology	-	-	-

The number of ESTs, the length of the sequence and the potential open reading frames (ORFs) are indicated. The 15 ESTs indicated in bold are homologous to known sequences. For these the best BLASTx match, the species, the E-value of the alignment, and the molecular function are indicated.

^a ^Selected for studying the expression in ovaries throught qRT-PCR

* From subtractive library assemble against cDNA ovary library

**Minimum length of 100 bp

The 34 ESTs resulting from assembling were compared against available databases in order to find similarities with known sequences. The ESTs were compared with non-redundant databases of all organisms, as well as of arthropods in particular. We carried out direct nucleotide comparison (blastn), followed by a dynamic translation comparison (blastx), and only matches with *E*-values lower than 10^-04 ^were considered significant for labelling them as known genes. Sequences with an *E*-value higher than 10^-04^, were labelled as undescribed. In summary, we found that 44% sequences had counterpart genes known in other organisms (Table 1) whereas 56% were undescribed. EST length ranged from 136 to 1616 base pairs (bp) in the case of known genes (Table 1), and from 140 to 2702 bp in the group of undescribed sequences.

The Geneious Pro 3.7 program was used to predict putative open reading frames (ORFs) in the 34 ESTs classified as unique sequences. Setting a minimal length of 100 bp and considering that the ORF covers most of the sequence as premises, results indicated that 29 out of 34 ESTs contain a putative ORF (Table 1).

### Gene Ontology analysis

**Figure 1:**
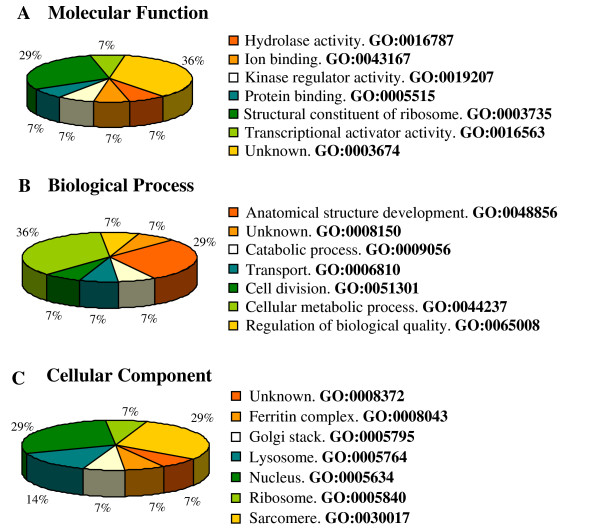
**Classification of ESTs according to Gene Ontology criteria**. Gene Ontology analysis was carried out on the post-vitellogenic genes isolated in the ovaries of *Blattella germanica *by SSH. Molecular Function (A), Biological Process (B) and Cellular Component (C).

Gene Ontology analysis of the 34 unique sequences was carried out using the GoAnna program for a general approach, and the FlyBase to classify them according to *D. melanogaster *labelling. Sequences were classified into the three ontology categories: "Molecular function", "Biological process" and "Cellular component" (Figure 1:A, B and 1C, respectively). In the category "Molecular function", the most frequent activity was *unknown *(36% of the sequences), which represents proteins with no associated functions, followed by *structural constituent of ribosome activity *(29%). The most frequent "Biological process" was *cellular metabolic process *(36%), followed by *unknown *(29%). Concerning the category "Cellular component", *ribosome *and *unknown *were the most frequent activities (29% each).

### Expression patterns

**Figure 2:**
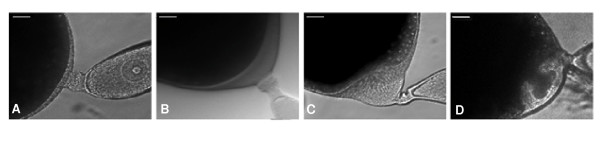
**Stages of chorion formation**. Apical pole of the basal oocyte of *Blattella germanica *ovaries in different stages of choriogenesis. A) day 7 just before the first symptoms of chorion secretion, B) day 7, early chorion stage (7EC), C) day 7, mid chorion stage (7MC), and D) late chorion stage (7LC). The process from 7EC to 7LC lasts 15 h approximately. Scale: 50 μm.

In order to assess the efficiency and quality of the SSH results, the expression pattern of a selection of genes was investigated by quantitative real-time PCR (qRT-PCR). Expression was studied in the ovary of adult females from emergence (day 0) to oviposition (day 7). Ovaries on day 7 were further subdivided into four stages, depending on the degree of chorion formation: 7, no signs of choriogenesis, with the apical pole of the basal oocyte showing the basement membrane very close to the follicular epithelium (Figure 2A); 7EC, early choriogenesis (EC), with the basement membrane of the apical pole slightly pulled away from the follicle cells (Figure 2B); 7MC, mid choriogenesis (MC), with the basement membrane of the apical pole showing a concavity (Figure 2C); 7LC, late choriogenesis (LC), with the basement membrane of the apical pole showing a claviform aspect (Figure 2D). The process from 7EC to LC lasts 15 h approximately[7].

Sequences selected to assess the efficiency of the SSH library were: Yellow-g, Bg30001, Bg30017, cuticular protein-like, follicle cell protein 3c, cathepsin-L and CG10407-PA-like. They represent known and undescribed sequences. Yellow-g is homologous of yellow-g CG5717-PA of *D. melanogaster*, which has an important role in vitelline membrane cross-linking [13]. Follicle cell protein 3c is homologous to follicle cell protein 3c CG4015-PA of *D. melanogaster*, which is involved in the formation of the vitelline membrane [14]. Cathepsin-L is homologous to cathepsin-L described in the decapod crustacean *Penaeus monodon *(best *E*-value) and other arthropods, where it participates in egg yolk degradation [15]. Cuticular protein-like has 26% identity with a hypothetical protein in the beetle *Tribolium castaneum *(XP_96686) and 20% of identity with cuticular protein CPG12 isoform 1 of the aphid *Acyrthosiphon pisum *(XP_001951490). CG10407-PA-like has 48% identity with CG10407-PA of *T. castaneum *(XP_973426), which contains domains typical of odorant binding proteins and juvenile hormone binding proteins. Among the undescribed sequences, we chose Bg30001 and Bg30017 because there are more than 20 ESTs of them in each contig, which suggests that the corresponding transcripts are particularly abundant in post-vitellogenic ovaries.

**Figure 3:**
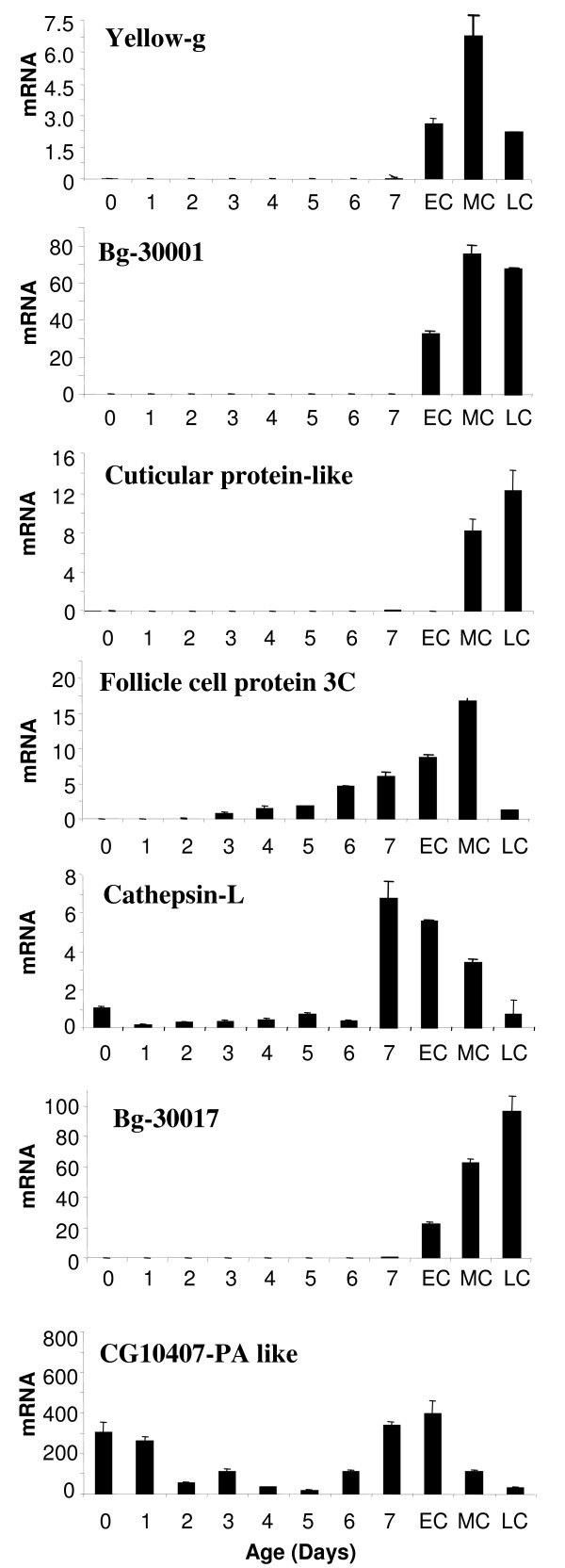
**Expression patterns of post-vitellogenic mRNA's isolated by SSH from ovaries of *B. germanica***. Expression pattern of seven post-vitellogenic genes in ovaries of adult *Blattella germanica *during the first gonadotrophic cycle. 7-day-old females in the period of chorion formation were divided into three stages: EC (early choriogenesis), MC (mid choriogenesis) and LC (late choriogenesis), according to criteria shown in figure 2. qRT-PCR was normalized against BgActin-5c. Data represent copies of mRNA per copy of BgActin-5c, and are expressed as the mean ± SD (n = 3).

Results (Figure 3) showed that the seven genes studied are expressed differentially during post-vitellogenesis, and all of them have maximal expression in some stage of choriogenesis. Yellow-g and cuticular protein-like, are expressed very transiently during choriogenesis, and their maximum expression is 6- and 12-fold higher than that of *B. germanica *actin-5c (BgActin-5c), respectively. The mRNA of follicle cell protein 3c appears in 3-day-old females, their levels increase slightly as basal oocyte matures, peak at MC, and suddenly decrease in LC. Cathepsin-L and CG10407-PA-like mRNAs are present during the entire gonadotrophic cycle, though with changing levels. Cathepsin-L show low mRNA levels in pre-vitellogenic and vitellogenic ovaries, they peak on day 7, just before the onset of choriogenesis, and then decrease progressively until LC. Those of CG10407-PA-like are relatively high at the beginning of the cycle, decrease during vitellogenesis (subtraction days) and increase during post-vitellogenesis, peaking on EC stage.

Bg30001 and Bg30017, which have no homologues in databases, are expressed transiently during choriogenesis, similarly as yellow-g and cuticular protein-like. Bg30001 expression is ca. 80-fold higher than that of BgActin-5c, and maximal mRNA levels occur at MC and LC (Figure 3). The expression pattern of Bg30017 is slightly different; mRNA levels increase progressively from EC to LC, where expression is ca. 100-fold higher than that of BgActin-5c (Figure 3).

### Localisation and function in the ovary

**Figure 4:**
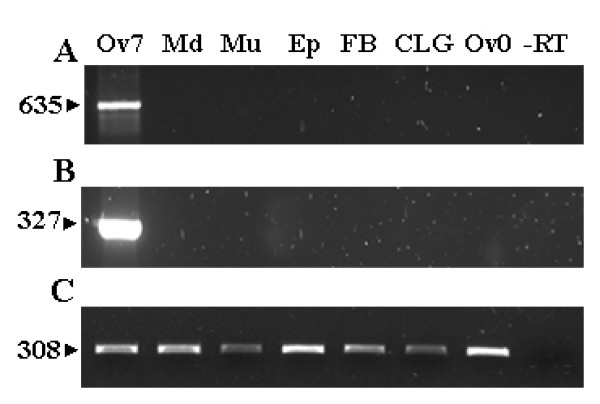
**Expression of Bg30001 and Bg30017 in different tissues**. mRNA of Bg30001 **(A) **and Bg30017 **(B) **in different tissues of *Blattella germanica *adult females. RT-PCR was carried out with total RNA isolated from 7-day-old adult ovaries in the period of chorion formation (Ov7), midgut (Md), muscle (Mu), epidermis (Ep), fat body (FB), colleterial gland (CLG), and from 0-day-old ovaries (Ov0), -RT (without reverse transcriptase) is shown as a negative control. BgActin-5c levels were used as a reference. Numbers on the left indicate the size of the amplified cDNA fragment (bp).

**Figure 5:**
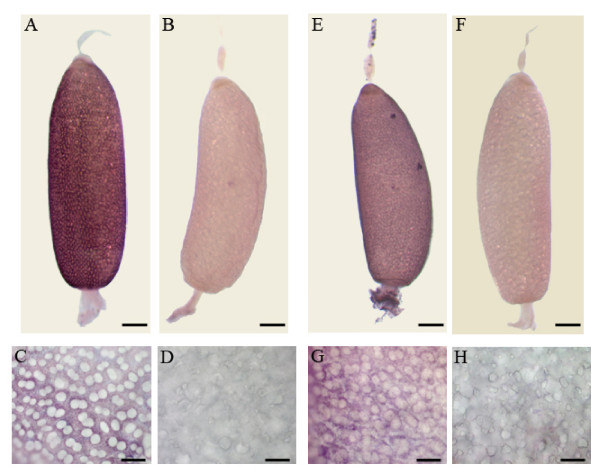
**Localization of Bg30001 and Bg30017 expression**. In situ hybridization using antisense Bg30001 and Bg30017 RNA probes. mRNA of Bg30001 **(A) **and Bg30017 **(E) **is detected in the follicular epithelium of basal oocytes from 7-day-old females in the period of chorion formation, but not in sub-basal oocytes or in the germarium. Detail of the follicular cells showing that Bg30001 **(C) **and Bg30017 **(G) **transcripts localize in the cytoplasm of the follicular cells. Control ovarioles tested with sense probes of Bg30001 **(B, D) **and Bg30017 **(F, H) **were not labelled. Scale bars: 200 μm in panels A, B, E, F; and 50 μm in panels C, D, G, H.

In order to assess whether Bg30001 and Bg30017 are specific from the ovary, we studied its expression by semiquantitative RT-PCR in different tissues of adult females. RNA from muscle, midgut, epidermis, fat body, colleterial glands and ovaries from newly emerged females and 7-day-old females were included in the study. As shown in figure 4, the expression of Bg30001 and Bg30017 is restricted to mature ovaries. Furthermore, in situ hybridization studies showed that both genes are specifically expressed in the follicular cells cytoplasm of basal oocytes during choriogenesis (Figure 5). Interestingly, neither the sub-basal oocytes nor the germarium were labelled, and optical sections of the basal oocyte showed that the ooplasm and the oocyte nucleus were also free of label. These spatial data and the temporal data afforded by expression studies strongly suggest that Bg30001 and Bg30017 are involved in the process of chorion formation.

Finally, we also tried RNAi experiments with the aim of assessing the specific functions of the post-vitellogenic genes found. Methodology was developed for yellow-g, given that its role in vitelline membrane cross-linking in *D. melanogaster *[13] suggested to us that RNAi phenotypes would be readily recognized and serve as a reference for studies with other genes. We used different doses of yellow-g dsRNA (1, 2.5, 5, 10 and 50 μg) and treated adult females of different ages (5-, 6- and 7-day-old), but we did not observe any phenotypic effect (Additional file 1). We presume that the very transient expression of yellow-g (as occurs in most genes from our SSH library) made impossible the completion of the sequential steps for RNAi.

## Discussion

### The Suppression Subtractive Hybridization library

The SSH approach has proven to be efficient for isolating genes specifically expressed after vitellogenesis in the ovaries of *B. germanica*. We obtained sequences of 34 genes, which give us the first cues to elucidate the molecular mechanisms regulating the final stages of oogenesis in panoistic ovaries, choriogenesis in particular. The expression pattern of seven of these genes in the adult ovary revealed that the highest mRNA levels generally occur after vitellogenesis (Figure 3), thus validating the SSH approach.

The SSH technique has been improved to equalize low and high abundant sequences, although a remarkable degree of redundancy has been found in three cases: the undescribed sequence Bg30017 (with 28 ESTs), the cuticular protein-like (with 52 ESTs) and the undescribed sequence Bg30009 (with 91 ESTs) (Table 1). In the case of Bg30017 and cuticular protein-like, the high number of reads could be correlated with very high mRNA levels during choriogenesis (Figure 2). High expression levels during choriogenesis is typical of chorion genes localized in specific genome regions which are overreplicated in order to facilitate high transcription rates in a short time [13].

### Sequences homologous to known genes

We have isolated 15 sequences (44% of the SSH library sequences) which are homologous to known gene sequences present in databases (Table 1). Among these 15 sequences, two proteins, namely follicle cell protein 3c and yellow-g, have been described as involved in insect eggshell formation [14,16-18]. In our expression studies (Figure 3), follicle cell protein 3c is expressed earlier than yellow-g, which suggests a function related to vitelline membrane formation. Nevertheless, the sequence of follicle cell protein 3c does not look like a typical eggshell protein, but rather appears more compatible with a protein with intracellular functions [14]. Yellow-g belongs to a gene family composed by 14 genes generally encoding secreted proteins related to Royal Jelly and restricted to insects [16,17]. In *D. melanogaster*, yellow-g is needed for proper egg formation, probably for catalyzing the cross-linking of eggshell layers [13], functions that have been postulated also in ants [18]. The structural similarity and the expression pattern, suggest that yellow-g may participate in the cross-linking of inner or outer chorion layers in *B. germanica*.

We have also isolated four ribosomal proteins: L18a, L24, L36e and L44e, which must be involved in translation and are obviously necessary for the synthesis of proteins in large amounts over a short period of time [19]. In *D. melanogaster*, the protein baiser is required for the secretion or removal of misfolded proteins during chorion formation and for the activity of maternal products in early embryogenesis [20]. Concerning troponin, Ono and Ono [21] identified it as a component of filaments in the sheath cells of the *C. elegans *ovary, being an essential regulator of cytoskeletal activity and playing also a role in ovarian contraction during ovulation. Similar functions for these proteins could be postulated in late oogenesis of *B. germanica*.

Other genes isolated in *B. germanica*, like cathepsin-L, ferritin 2 and cyclin b, may have significant roles during embryogenesis, as suggested by previous reports on homologous genes in other species. Cathepsin-L is a cysteine proteinase belonging to papain family, which is expressed in lysosomes [22]. The role of cathepsin-L has been widely studied in mammals, where it is involved in oocyte development, ovulation and follicular atresia [23]. In arthropods, Fagotto and colleagues [15] reported that cathepsin-L is the major yolk cysteine proteinase, and that it is stored as a latent proenzyme in tick eggs, whereas Nordin and colleagues [24] described it as the major vitellin proteolytic enzyme in *B. germanica*. These reports suggest that cathepsin-L is more related with egg yolk metabolism than with oocyte development, although the expression pattern showing maximal mRNA levels on day 7 (Figure 3) also suggests a function related with follicular epithelium degradation (which occurs in parallel to choriogenesis). Another identified gene is that coding for ferritin 2. Ferritins are iron storage proteins that protect cells of free iron (which can be highly toxic), but also made this metal readily available. In fishes, high levels of ferritin mRNAs have been observed in the ovary [25], but little is known about its specific role. Georgieva and colleagues [26] reported that ferritin messages and proteins are synthesized in the ovary, stored in the eggs and used by the developing embryo, although the function during oogenesis remains unclear. Cyclin B, along with cyclin A and B3, can form complexes with cyclin-dependent protein kinase 2. They act as mitotic cyclins, which are needed for blocking DNA re-replication and for promoting the onset of mitosis [27]. Dalby and Glover, reported that transcripts of cyclin B are expressed at the posterior pole of *D. melanogaster *oocytes at a late stage of oogenesis, being incorporated into the developing embryo [28]. According to these data, the isolation of cyclin B at the end of oogenesis in *B. germanica *suggest that it is a maternal gene product having a role in embryogenesis, for example in microtubule dynamics as demonstrated in the embryo of *D. melanogaster *[29].

### Novel genes

From the SSH, we also isolated 186 ESTs which clustered in 19 unique sequences without homology to sequences in databases. The generally short length of cloned cDNAs (Table 1) derives from the construction procedure of the SSH library, in which the double stranded cDNAs are digested with the enzyme *Rsa*I to ligate an adaptor for PCR amplification. Apparently, this restriction site is very frequent in the genome [30], thus promoting the formation of short fragments, which makes comparisons with databases more difficult. This circumstance can account for the relatively high percentage of undescribed sequences encountered. Another reason for explaining this high percentage may be that some ESTs could correspond to 3' or 5' untranslated regions (UTRs), which makes impossible the finding of homologues in protein databases.

However, it is likely that most of these undescribed sequences correspond to novel or orphan genes, in the sense of Albà and Castresana [31]. For example, Bg30001 and Bg30017, in spite of having a sequence fragment long enough (more than 1000 bp each) to establish safe comparisons, have no homologues in databases. These two characteristic novel genes were taken as examples for studying not only the pattern of expression by qRT-PCR (Figure 3), but also for examining them in different tissues by RT-PCR (Figure 4) and for localizing their expression in the ovary by in situ hybridization (Figure 5). Results indicated that both are specifically expressed after vitellogenesis in the cytoplasm of basal oocyte follicular cells, which strongly suggests that they play a role in chorion formation.

Functional studies with RNAi taking yellow-g as model were unsuccessful, in spite of the different doses and different ages tested (Additional file 1). Given that other genes have been successfully disrupted by RNAi in the ovary of *B. germanica *[32,33], we presume that negative results obtained here are due to the very transient expression of yellow-g, which do not permit the completion of the RNAi steps that lead to transcript degradation. This might also occur with other genes from our SSH library which have similar transient pattern of expression, although systematic RNAi experiments for all 19 undescribed sequences are currently being attempted in our laboratory.

In any case, these 19 novel sequences seem to be exclusive of panoistic ovaries, which points to the possibility that the molecular basis of choriogenesis in panoistic ovaries might be quite different from that of meroistic ones. Indeed, our SSH library did not reveal any of the typical chorion genes belonging to chorion protein families A, B or C of moths; or vitelline membrane proteins (like sV17, sV23, sV26), chorion proteins (like s16, s18, s15, s19, s36 and s38) or smaller chorion proteins of flies [3,8]. The fact that *B. germanica *package the eggs into an ootheca, could also account for the differences. Comparative genomics may be an efficient approach to compare putative chorion genes in meroistic and panoistic ovaries, or to compare panoistic species which protect the eggs in an ootheca with those laying free eggs. This seems to be the fairest way to illuminate the mechanisms underlying the transition from one type to another.

## Conclusion

Our results show that the SSH approach was appropriate to identify ovarian genes specifically expressed at the end of vitellogenesis and involved in choriogenesis in *B. germanica*. We obtained sequences of 34 genes, 44% of which had homologues in other organisms, whereas 56% had no significant similarity to any of the database entries. For most of the 44% labelled genes, functions related to choriogenesis can be predicted. This is the case, for example, of yellow-g and follicle cell protein 3c, which are well described as involved in chorion formation in insect meroistic ovaries. The high percentage of undescribed genes and the clamorous absence of most typical chorion genes of meroistic ovaries available in databases suggest that molecular mechanisms underlying choriogenesis in panoistic ovaries are significantly different from those of meroistic ones.

## Methods

### Insect sampling

Specimens of *B. germanica *were obtained from a colony reared in the dark at 30 ± 1°C and 60–70% r.h. Freshly ecdysed adult females were selected and used at appropriate ages. The length of the basal oocyte was used to stage the ovaries from 0- to 7-day-old, whereas the stages of choriogenesis (EC, MC or LC) were determined according to the morphology of the apical pole of the basal oocyte (Figure 2). All dissections and tissue sampling were carried out on carbon dioxide-anaesthetized specimens.

### RNA extraction

To construct SSH libraries, total RNA was isolated using RNeasy^® ^Plant Mini Kit (Qiagen). PolyA^+ ^mRNA was obtained using Dynabeads Oligo (dT) (Dynal Biotech ASA), following the manufacturer's protocols. For mRNA expression studies, total RNA was isolated from four to six ovary pair pools obtained in chosen ages and stages of the first gonadotrophic cycle, using the Gen Elute Mammalian Total RNA kit (Sigma). An amount of 500 ng of each RNA extraction was treated with DNAse (Promega) and reverse transcribed with Superscript II reverse transcriptase (Invitrogen) and random hexamers (Promega). RNA quantity and quality was estimated by spectrophotometric absorption at 260 nm in a Nanodrop Spectrophotometer ND-1000^®^.

### Suppression subtractive hybridization (SSH) library

SSH was carried out using the PCR-select cDNA Subtraction Kit (Clontech), following the manufacturer's protocols. The tester library (post-vitellogenic ovaries) was prepared with 1.0 μg of polyA^+ ^mRNA from a 10 ovary-pair pool from 6- to 7-day-old females with fully chorionated oocytes. The driver library (vitellogenic ovaries) was prepared with the same amount of polyA^+ ^mRNA from 3- to 4-day-old females. Subtractive PCRs were carried out after 32 cycles of primary PCR and 17 cycles of secondary PCR with AccuTaq-LA DNA Polymerase (Sigma-Aldrich).

### Cloning and sequencing

Blunt-end PCR products were cloned in pCR^®^-Blunt II-TOPO plasmid vector (Zero Blunt TOPO PCR Cloning Kit; Invitrogen) following the manufacturer's recommendations. Cloning efficiency was evaluated with PCR amplifications of bacterial colonies selected at random with vector specific primers and agarose gel analysis, resulting that 98% of colonies having inserts of variable size. Therefore, 384 colonies were picked up at random, plated in LB-ampicilin medium in a 384 well plate and cultured overnight. Frozen plates were sent to Max Planck-Institut für Molekulare Genetik (Germany), for massive sequencing.

### DNA sequence analysis

The vector was trimmed using the ChromasPro program version 1.33, and the sequences were filtered for polyA^+ ^tail, for size (less than 100 bp), for doubles or for sequences with low quality read. To obtain unique sequences we made an assemble of the ESTs using the CAP3 program [34]. Unique DNA sequences were compared against non-redundant nucleotide and protein databases using BLAST 2.2.6 program [35]. Searches for potential open reading frames (ORF) were carried out using Geneious Pro trial program 3.7 [36]. We considered fair putative ORFs if they covered most of the sequence and had a minimum of 100 nucleotides. Classification of sequences was performed under Gene Ontology (GO) criteria. For a general approach, we used the GoAnna program (agbase.msstate.edu/GOAnna.html) using a similarity search [37], then we used the FlyBase to have the nomenclature of *D. melanogaster *as a reference.

### Semiquantitative RT-PCR

To study the expression of Bg30001 and Bg30017 in different tissues by semiquantitative RT-PCR, total RNA was isolated from muscles, fat bodies, epidermis, colleterial glands and midguts obtained from a pool of 6 adult females of different ages. An amount of 300 ng of each RNA extraction was treated with DNAse (Promega) and reverse transcribed with Superscript II reverse transcriptase (Invitrogen) and random hexamers (Promega). Samples were subjected to PCR amplification with 40 cycles at 94°C for 30 sec, 59°C for 30 sec, and 72°C for 40 sec. Primers used for Bg30001 and Bg30017 detection were as follows: forward (Bg30001F2), 5'-TTCCAAATACCCTGGATTCCC-3'; and reverse (Bg30001R2), 5'-ATGAATTTGAGTAGTACTAGT3'; forward (Bg30017F1), 5'-CCCCTGGGTTCCATATTACC-3'; and reverse(Bg30017R2), 5'TTTGATGCCTCCATGTTCAA-3'. As a reference, the same cDNAs were subjected to RT-PCR with a primer pair specific of BgActin-5c: forward (AcF1), 5'-TCGTTCGTGACATCAAGGAGAAGCT-3' and reverse (AcR1), 5'-TGTCGGCAATTCCAGGGTACATGGT-3'.

### Determination of mRNA levels with quantitative real-time PCR

PCR primers used in qRT-PCR expression studies were designed using the Primer Express 2.0 software (Applied Biosystems) (Table 2), in order to select an optimal primer annealing temperature of 59°C (58–60°C range). Expression of BgActin-5c (AJ862721) was used as a reference (primers indicated in Table 2). The efficiency of each primer set was first validated by constructing a standard curve through four serial dilutions. PCR reactions were carried out in triplicate in an ABI Prism 7000 Sequence Detection System^® ^(Applied Biosystems), using SYBR^®^Green (Power SYBR^® ^Green PCR Master Mix; Applied Biosystems). A control without template was included in all batches. The PCR program began with a single cycle at 95°C for 10 min, 40 cycles at 95°C for 15 s and 60°C for 60 s. Afterwards, the PCR products were heated to 95°C for 15 s, cooled to 60°C for 15 s and heated to 95°C for 15 s, in order to measure the dissociation curves and to determine an unique PCR product for each gene. mRNA levels were calculated relative to BgActin-5c expression using the ABI Prism 7000 SDS Software (version 1.2.3). We followed a method based in Ct (threshold-cycle) according to the Pfaffl mathematical model [38], simplifying to 2^ΔΔCt ^because the calculated efficiency values for studied genes and BgActin-5c amplicons were always within the range of 95 to 100%; therefore, no correction for efficiency was used in further calculations. Results are given as copies of mRNA per 1,000 copies of BgActin-5c mRNA.

**Table 2:** Primers used for qRT-PCR. F: Forward, R: Reverse

**Primer name**		**Primer sequence**	**Amplicon length**
Bg Follicle cell protein 3-c	F	5'TAGTCCAGATCCCCCTAAGGG 3'	51 bp
	R	5'CCTTCTGCATGAGCTGATGGA 3'	
			
Bg Yellow-g	F	5'ACTGACACATCCTTCAAGCATGA 3'	52 bp
	R	5'ACTGACACATCCTTCAAGCATGA 3'	
			
Bg Cathepsin-L	F	5'AGAGACTGGTGAAGAGTACTGGCTAGT 3'	51 bp
	R	5'AGTTAAAAATTCATGGGGTACAACTTG 3'	
			
Bg CG10407-PA-like	F	5'GTTCGCCTGGACAACCTTTTC 3'	51 bp
	R	5'CAAAAAACTTGGTGACGCAATG 3'	
			
Bg 30001	F1	5'TCGTGCTTTTCAATGTGCGTA 3'	51 bp
	R1	5'GGGAATCCAGGGTATTTGGAA 3'	
			
Bg Cuticular Protein-like	F	5'GGCTCTCGTCGTTTACACCG 3'	51 bp
	R	5'ACCAAGCGAGAAATAGCGGA 3'	
			
Bg 30017	F1	5'CCCCTGGGTTCCATATTACC 3'	71 bp
	R1	5'TTCCAAGAAGACCTGGCAGT 3'	
			
Bg Actin-5c	F	5'AGCTTCCTGATGGTCAGGTGA 3'	213 bp
	R	5'ACCATGTACCCTGGAATTGCCGACA 3'	

### In situ hybridization

For in situ hybridization studies of Bg30001 and Bg30017 in the ovary, digoxigenin-labeled RNA probes (sense and antisense) were generated by transcription in vitro using SP6 or T7 RNA polymerases (Promega) and DIG RNA labeling mix (Roche). Bg30001 and Bg30017 probes were obtained from sequence fragments of 213 bp and 327 bp, respectively. Ovarioles were dissected under PBS 0.2 M at pH 6.8, and fixation, hybridization and detection steps were carried out essentially following the method reported [39]. Samples were mounted in Mowiol medium (Calbiochem) and examined under a stereomicroscope Discovery.V8.Stereo (Zeiss) and a microscope AxioImager.Z1 (Zeiss).

## Authors' contributions

PI carried out the SSH library and molecular genetic studies, participated in the sequence analysis and drafted the manuscript. XB participated in the design of the study and contributed to write the manuscript. MDP conceived the study, participated in its design and coordination and wrote the manuscript. All authors read and approved the final manuscript.

## Supplementary Material

Additional file 1 **RNAi of yellow-g in adult *Blattella germanica***. Methods used and results obtained in RNAi experiments to silence BgYellow-g. Click here for file
