# Supplementary material for: Identifying genes related to choriogenesis in insect panoistic ovaries by Suppression Subtractive Hybridization
Source: BMC Genomics. 2009 Apr 30;10:206. doi: 10.1186/1471-2164-10-206 (PMC2683872; doi:10.1186/1471-2164-10-206)
Supplement: Additional file 1 — RNAi of yellow-g in adult Blattella germanica. Methods used and results obtained in RNAi experiments to silence BgYellow-g. [file 1471-2164-10-206-S1.doc]

# Additional File 1

# RNAi of yellow-g in adult *Blattella germanica*

# Methods

## Double-stranded RNA synthesis and treatments

To obtain a double-stranded RNA to target BgYellow-g mRNA (dsBgYellow-g) a 575 bp fragment was amplified by PCR and subcloned into the pSTBlue™-1 vector. The primers used in the design of dsBgYellow-g were as follows: 5′- GATGCTCAAGATATCCTTTGGA-3′ and 5′- ACTGACACATCCTTCAAGCATGA-3′. As control dsRNA, we used a 92 bp noncoding sequence from the pSTBlue-1 vector (dsControl). The double-stranded RNA were synthesised as previously described [1, 2]. A final volume of 1 μl containing the chosen dose was injected into the abdomen of females representing three ages in the first gonadotrophic cycle. Controls were injected with the same volume and dose of dsControl.

# Results

## RNAi studies

5-, 6- or 7-day-old adult females of *Blattella germanica* were injected with different doses of dsBgYellow-g or dsControl, but no differences were observed between the treated and the control group in terms of oocyte maturation, embryo development and egg viability (Table 1). mRNA levels were studied in 7-day-old dsBgYellow-g-treated females and did not give significant differences with respect to controls (Figure 1).

## Table 1. Age at oviposition, time of ootheca transport and nymphs emerged from the ootheca in dsBgYellow-g-treated specimens and in controls of B. germanica. Results are expressed as the mean ± SD. ND: Not determined (most oothecae resulting from 7-day-old treated females were dissected to examine the embryo phenotype or to measure mRNA levels of yellow-g).

| **Age of treatment (days)** | **Treatment** | **Dose (μg)** | **n** | **Age at oviposition** | **Time of ootheca transport (days)** | **Nymphs emerged** |
| --- | --- | --- | --- | --- | --- | --- |
| 5 | dsControl | 1 | 15 | 8 | 16.7 ± 0.5 | 31.5 ± 7.7 |
|  | dsBgYellow-g |  | 17 | 8 | 16.5 ± 0.5 | 31.8 ± 8.8 |
|  | dsControl | 5 | 24 | 8 | 18 ± 0 | 38.4 ± 6.2 |
|  | dsBgYellow-g |  | 22 | 8 | 16.7 ± 1.2 | 35.5 ± 5.8 |
|  | dsControl | 10 | 5 | 8 | 16.8 ± 1.2 | 38 ± 13.5 |
|  | dsBgYellow-g |  | 6 | 8 | 17.4 ± 2 | 33.2± 11.4 |
|  | dsControl | 50 | 10 | 8 | 16.8 ± 0.9 | 38.1 ± 5.9 |
|  | dsBgYellow-g |  | 14 | 8 | 16.8 ± 0.8 | 23.8 ± 11.8 |
| 6 | dsControl | 2.5 | 6 | 8 | 16.3 ± 0.8 | 37 ± 6 |
|  | dsBgYellow-g |  | 6 | 8 | 16.6 ± 1.6 | 41.6 ± 4.5 |
|  | dsControl | 5 | 12 | 8 | 16.2 ± 0.9 | 32.7 ± 8.3 |
|  | dsBgYellow-g |  | 18 | 8 | 16.6 ± 1.1 | 36.3 ± 9 |
| 7 | dsControl | 2.5 | 4 | 8 | 19.3 ± 0.9 | ND |
|  | dsBgYellow-g |  | 14 | 8 | 19.7 ± 0.6 | ND |
| 7 | dsControl | 5 | 6 | 8 | 16 ± 0 | ND |
|  | dsBgYellow-g |  | 10 | 8 | 17 ± 0.8 | ND |

0

1000

2000

3000

4000

5000

6000

dsControl

mRNA relative expression

dsBgYellow-g

**MC**

**LC**

## Figure 1: mRNA levels of yellow-g in ovaries of Blattella germanica treated with dsBgYellow-g and dsControl. Fresh 7-day-old females were injected with 5 μg of dsBgYellow-g or dsControl. Ovaries were dissected at mid choriogenesis (MC) or at late choriogenesis (LC). Levels of yellow-g mRNA were quantified by qRT-PCR analysis. Not significant differences were found between controls and treated. qRT-PCR was normalized against actin-5c. Data expressed as the mean ± SD (n=3 in all cases).

# Conclusion

A number of ovarian genes have been previously and successfully interfered by RNAi in adult *B. germanica*, for example those of vitellogenin receptor [1] and lipophorin receptor [2]. The present negative results might be explained by the very transient expression of yellow-g, which made impossible the successful completion of the sequential steps for RNAi, and the disruption of gene expression.

**References**

1. Ciudad L, Piulachs, M.D., Bellés, X.: **Systemic RNAi of the cockroach vitellogenin receptor results in a phenotype similar to that of the Drosophila yolkless mutant.** *FEBS Journal* 2006, **273**(2):325-335.

2. Ciudad L, Belles X, Piulachs MD: **Structural and RNAi characterization of the German cockroach lipophorin receptor, and the evolutionary relationships of lipoprotein receptors**. *BMC Mol Biol* 2007, **8**:53.
